# Analysis of self-perceived use of spectrum of teaching styles in Italian physical education teachers

**DOI:** 10.3389/fspor.2024.1397511

**Published:** 2024-06-11

**Authors:** Domenico Monacis, Francesca Latino, Cristina d’Arando, Matteo Bibba, Sabrina Annoscia, Giacomo Pascali, Italo Sannicandro, Dario Colella

**Affiliations:** ^1^ Department of Wellbeing, Nutrition and Sport, Pegaso Telematic University, Naploli, Italy; ^2^ Department of Psychology and Education, Pegaso Telematic University, Naples, Italy; ^3^ Department of Humanities. Letters, Cultural Heritage, Education Sciences, University of Foggia, Foggia, Italy; ^4^ DiSU, Department of Humanities, University of Basilicata, Potenza, Italy; ^5^ Department of Literature, Languages and Cultural Heritage, University of Cagliari, Cagliari, Italy; ^6^ Department of Biological and Environmental Sciences and Technologies, University of Salento, Lecce, Italy; ^7^ Department of Clinical and Experimental Medicine, University of Foggia, Foggia, Italy

**Keywords:** quality physical education, school children, spectrum of teaching styles, teacher-student relation, teacher’s behavior

## Abstract

**Introduction:**

The present study aims to assess PE teachers' perception about the use of teaching styles during curricular lessons.

**Methods:**

The sample involved a total of 83 PE teachers (M = 41, F = 42, main age = 43,74 ± 10,76) divided according to years of service (0–4 = 36%, 5–10 = 34%, and over 10 = 30%) and academic training (Higher Institutes of Physical Education = 44% and master's degree = 56%). Teachers complete a digitalized version of a self-produced questionnaire to assess how many times they use each teaching styles during the last month.

**Results:**

Results show that (a) reproduction rather than production teaching styles were often used, while reproduction teachings styles were most frequently used regardless of years of service.

**Discussion:**

Future research should investigate PE teacher's behavior to enhance the quality of physical education in school.

## 1 Introduction

Teaching motor skills requires the use of multiple teaching styles (1) to promote personalized teaching interventions through the proposal of specific executive variants and organizational modalities of motor tasks. It is necessary to recognize that educational research and good practice in the field of physical education (PE) and physical activities have made significant progress and developments deepening the disciplinary analysis in different contexts and educational settings. In fact, findings have highlighted that motor tasks and the relative adaptations to individual differences represent the starting point from which to propose new didactic paths for motor skills learning (2). On the contrary, there is a need to broaden and verify with methodological accuracy the studies based on the analysis of the teacher-student relathionship, and on the organization of groups and spaces/environments to promote different ways of learning in students. The following paper presents the preliminary results of a study carried out in secondary school aimed at detecting and interpreting the teaching styles mainly used by a sample of teachers during physical education lessons.

### 1.1 Theory of motor learning. Brief review

Findings in the field of motor learning processes suggests the importance of the body-motor experiences experienced by the child in structured contexts (family, school, sport initiation, etc.) and unstructured (after-school, equipped spaces, etc.) in which to be physically active (3). Recent theories of motor learning, in fact, exceed previous theoretical models and paradigms based on the standardized and well-defined sequence of phases that attribute to each age (or stage of learning) specific and predefined expected behaviors. For example, Fitts and Posner (4) describes the process of motor learning as the transition from the cognitive stage (what to learn) to the autonomous one (when and why to perform a certain movement), passing through the associative stage (how to perform the movement you are learning), while, according to Meinel and Schnabel (5) the motor learning process involves the succession of three phases, such as raw coordination, refined coordination and variable availability, characterized by the progressive improvement not only in terms of acquisition of motor skills, but also automation of gestures and movements. Schmidt (6), on the other hand, applies the concept of schema (proper to psychology) to define the generalized motor program as a class of actions that have similar structural characteristics. Learning, therefore, is understood as the result of a general program that, through adaptation to new situations and the variability of the surrounding environment, offers different opportunities for movement. In addition, the main theoretical approaches that dealt with the definition and description of learning processes and motor control—behaviorist, cognitive and ecological-dynamic—differ mainly in the ways in which they consider the relationship between perception and action in the execution of the movement. The behaviorist approach considers, in fact, motor learning as the response to a given external stimulus and attributes, therefore, great importance to behavior (product) rather than to cognitive-mental activity (process) (7, 8).

On the contrary, the cognitive model considers the individual as an active, vigilant, and motivated entity for learning, and defines movement as the result of general cognitive schemes designed to guide or generate motor acts aimed at a purpose (9). In this model, perception represents the starter of any movement, followed, in order, by decision and any voluntary motor action. The succession of perception—decision—action presupposes the continuous recall from the memory of previous motor experiences to recover, compare and choose the best and most appropriate option to the specific situation among the different possible solutions (10).

According to the ecological-dynamic approach, instead, perception is considered a process through which the individual, without using what is contained in memory systems, identifies, discovers and experiments in the environment and from the environment a whole series of information functional to the execution of a given movement; this approach is also defined ecological since it considers the complex interaction between individual-task-environment (11). The decision, therefore, comes from the interaction between the person, the movement, and the environment—without necessarily resorting to memory systems—and, therefore, takes on meaning only within the perception-action association. According to the theory of dynamic systems, in fact, learning is understood as the direct result of the interaction of the individual with the surrounding environment and, this aspect, finds its highest representation and expression in the connection between perception-action (12).

The conditions dictated by the learning environment, therefore, offer to the students' different opportunities for action and the possibility of performing multiple variations of a given movement that are adapted to different situations.

The variability of the motor responses of children is conditioned by the situation (near-far, large, small field, heavy-light ball, number of players on the field, etc.) already present in the environment or in school setting, intentionally proposed by the teacher.

The model of the Constraints-Led Approach (CLA) is based, in fact, on the modification of the constraints and variants of the movement—in relation to the motor task, the environment and the ability of the performer—to encourage self-regulation and the implementation of the most effective motor solutions, with important implications in the field of education and sports (for example related to the ability to solve problems or make decisions) (13, 14). In this regard, international literature has also highlighted how non-linear educational approaches characterize the spontaneous learning of motor skills by the child, in which the teacher is assigned the role of “guide” which intentionally orients students towards the discovery and resolution of some motor problems (15, 16).

Such an approach is expressed in the research, by the child and the teacher, of dynamic and variable learning contexts, able to enhance the body-stimulus/ external-environment relationship in order to guide decision-making processes (what to do) and problem solving (how to do?) towards the definition of a certain movement, leaving the possibility for the child to experiment, try, make mistakes and try a series of tasks and activities that are not predefined, but open, adapted and customized to the individual motor abilities and skills (17, 18).

From this perspective, the learning process takes on a more global and inclusive connotation, closely linked to the opportunities and constraints that the environment offers and resulting from the reciprocal interactions between motor activity (e.g., motor task), child and environment (15).

### 1.2 Neuroscience, teacher's reflective behavior and motor learning in physical education

Recently the fields of intervention and the activities that make up the disciplinary structure of physical education in the school, have undergone major revisions and organizational updates and a significant expansion, assimilating—sometimes in a hasty way—the repertoire of contents and activities proposed to students with motor skills, object of learning. If this is accurate in terms of the relationship between disciplinary content and learning objectives, it is not always accurate in terms of the learning processes that are required and solicited by motor experiences.

In fact, the teaching of motor skills necessarily takes place through a wide repertoire of contents and organizational methods but develops and proceeds further, since it will have to tend to mobilize the different factors that structure the motor competence itself. This will be possible using different teaching styles that will allow to promote the didactic mediation and the learning process of the student (19–22). In a teaching process, lesson, learning unit, curriculum, modulation, variation, and interaction of teaching styles determines different ways of information processing and response by the student, allowing different and personalized learning methods and a non-linear pedagogical-didactic approach (15).

Recent evidence in the field of educational neuroscience have opened the way to new didactic-methodological reflections, giving increasing importance to how to teach the brain (learning) (23). In addition, Gola (24) underlines the pedagogical value of educational neuroscience: it is not only the learning experiences experienced by the child, but also, and above all, the quality and way the child learns to influence brain plasticity and cognitive development.

### 1.3 The Spectrum of Teaching Styles: an overview

The model of Teaching Styles (Spectrum Teaching Styles) proposed by Mosston and Ashworth (1) is a current and important methodological reference to address the complexity of motor competence and to study the interactions between the teacher and the group-class, the degree of responsibility and educational decisions. Therefore, the integration of quantitative (i.e., daily motor activity) and qualitative (i.e., contents, experiences, and modes of teacher-student relationship) variables of human movement are unavoidable for conducting and promoting research in physical education.

The Spectrum was presented as a unifying framework, a denominator to delineate teaching styles and it included 11 teaching styles: Command style–A, Practice Style-B, Reciprocal Style-C, Self-Check Style-D, Inclusion Style-E, Guided Discovery-F, Convergent Discovery Style-G, Divergent Discovery Styles-H, Learner Designed Individual Program Style-I, Learner Initiated Program Style-J, and Self-Teaching Style-K. The fundamental reference point regarding the variation of decisions from teacher to student. According to this conceptual framework Mosston and Ashworth (1), through the definition of teaching styles, present the transition from teaching in which the teacher expresses the highest degree of responsibility and decisions, in the choice of activities and executive and organizational methods (e.g., in athletics or gymnastics), to an approach in which, on the contrary, decisions and choices involve the student in the foreground (e.g., in bodily expression, activities in the natural environment; etc.).

Therefore, according to the lesser or greater students' decision-making autonomy, teaching styles are classified into reproduction styles (ranged from styles A to E) and production styles (ranged from F to K). Below a brief description of each teaching styles (Table 1).

**Table 1:** Reproduction and production teaching styles.

Reproduction teaching styles		Production teaching styles	
Command Style-A	Teacher takes all decisions (i.e., difficulty, duration, series, repetitions, use of tools, etc.) and students perform the task according to teacher's instructions. Students are asked to reproduce a specific performance or response proposed by teacher.	Guided Discovery-F	Teacher defines subject matter, target concept and questions sequential design asked to students. Students make decisions about certain subject within the topic designed by teacher and try to discover the predetermined motor answers.
Practice Style-B	Teacher prepares the organizational methods: individual tasks, in pairs, in groups, relay, paths, circuits, games. The teacher starts the learning process (or consolidation) of a motor skill by proposing the task in easy conditions, the number of executive variants is modulated according to the stage of the learning process (i.e., jump with the cord on equal feet joined, perform a flip forward, etc.). Performance difficulty, repetitions, duration, use of a tool are defined by the teacher. Students individually practice a memory/reproduction task while teachers provide private feedback.	Convergent Discovery Style-G	Teacher, as well as determine subject matter decision and target concepts to be discovered, designs specific questions to students allowing them to discover the correct way of performing a motor task. Students are asked to reason, question, and make logic-sequential connection to discover the correct motor answer.
Reciprocal Style-C	Students are asked to work together and in partnership, and teacher provide criteria of successfulness. Two students work together on a motor task proposed by teacher. One student performs the task while the other gives feedback. The motor and observation times alternate. The students practice in pairs at the same time.	Divergent Discovery Styles-H	Teacher makes decisions about the subject matter topic and specific questions to ask students. Students have to discover different and multiple solutions/motor answers to a question/situation posed by teachers.
Self-Check Style-D	Teacher provides a sheet designed of motor skills (e.g., jumping with the cable, throwing the ball with one hand to a fixed target, etc.), according to predefined criteria. The students work independently and self-check their performance according to the criteria given by teacher.	Learner Designed Individual Program Style-I	The teacher chooses a disciplinary field, but it is the student who makes most of the decisions about his/her motor experience. The student decides what he wants to learn within the teacher's programming, and then presents a personal motor sequence with the teacher's supervision.
Inclusion Style-E	Teacher identifies a motor task /activity, among the disciplinary areas, in which there are different levels of difficulty implying the proper use of executive variants (near-far; high-low; far-close; heavy-light; to one or two hands). The teacher proposes the different levels of difficulty for each student/sub-group and students may decide to perform the easiest or most difficult task by varying executive levels thus integrating the motor skills already learned.	Learner Initiated Program Style-J	Students decide the disciplinary area, e.g., group games with small tools. The teacher provides the basic executive criteria, but the student and the group are/are responsible for the organization and conduct. The teacher, if necessary, can help the group through feedback.
		Self-Teaching Style-K	Students decide completely the aim of learning, a new field/ theme researching insights and experiences to be performed and learned.

Recently, the analysis of teaching in physical education concerned the self-assessment of teaching styles, with particular reference to the frequency with which some of them were used (25). Findings revealed that teacher's perception differed according to personal knowledge and competence related to using teaching styles (26). Furthermore, the greater or lesser use of a specific teaching style is significantly related to teachers' belief about the style (27). A study conducted on 156 PE teachers in Turkey (28) reveals the prevailing use of reproduction (teacher-centered) teaching styles both in public and private schools. Moreover, another study investigated the frequency of use of each teaching styles in a sample of 110 Senior PE teachers (11–12 years of service). Results revealed that the most used teaching styles were Practice (about 94.5%), Command (77%) and Divergent Discovery (73.6%), while Self-Teaching (13.6%), Learned Initiated Program (21.8%) and Inclusion (47.2%) were the least used (25). However, other research highlighted the limited used of all (or almost all) the Spectrum of Teaching Styles during PE lessons (27).

## 2 Materials and methods

### 2.1 Aims

To the best of our knowledge, the present study is the first that aims to assess Italian PE teachers' perceived use of teaching styles during curricular lessons.

The following research questions arise:

(i) Can years of service and academic training determine different perception of teaching styles during PE lessons?
(ii) Does the perceived use of teaching styles differ significantly during PE teachers practice?

### 2.2 Participants

The sample was recruited from high school PE teachers involved in the Regional Observatory of Motor Development and Health Behavior in Apulia Region. From a total of 120 PE teachers, 90 were randomly enrolled in the present study of which 7 have decided not to participate in the study. The final sample involved 83 PE teachers (M = 41, F = 52, main age = 43,74 ± 10,76) divided according to years of service (0–4 = 36%, 5–10 = 34%, and over 10 = 30%) and academic training (Higher Institutes of Physical Education = 44%; Motor and Sports Science Degree = 56%).

### 2.3 Procedure and assessment

Teachers complete a digitalized version of a self-produced 11 items questionnaire to assess how many times they use each teaching styles during the last month. (i.e., “during the last month, how many times did you use the following teaching styles during PE lessons?”), and they were asked rate frequency with the following options: never (0 times a month); rarely (1–3 times a month); sometimes (4–6 times a month); often (7–9 times a month); almost always (over 9 times a month). Didactic instrument with a short description of each teaching styles and practical examples were also given to participants before answering the questionnaire. Informed consent to ensure participants’ voluntary participation and anonymity were obtained from all participants involved in the study. Data collection was carried out from January to June 2023.

### 2.4 Statistical analysis

After collecting data, the Chi-Quadro analysis was performed on the total sample and in relation to length of service (0–4 years, 5–10 years, and >10 years) and training (Higher Institutes of Physical Education or Graduates in Motor and Sport Sciences) to assess significant differences in the frequency of use of each style. The Yates correction was used for frequencies below 5. A contingency coefficient (C) was also calculated to assess the relationship between the nominal categories (years of service and academic training) and the frequencies of usage for each teaching style. Furthermore, data from questionnaire responses were analyzed on total sample, independently of academic training and years of service The significance index was set for *p* < .05 values. The statistical analysis was performed with SPSS 25.00 for Windows (Chicago, IL, USA).

## 3 Results

Frequency and percentage with which each teaching styles have been used by participants are reported to provide description of results.

The Table 2 highlights the significant differences in the use of teaching styles according the two attributes (years of service and academic training). Although no significant differences have been detected in relation to years of service, academic training appears to be significantly associated with the use of command styles, practice, inclusion, and guided discovery.

**Table 2:** Differences among the use of reproduction and production teaching styles according to years of service and academic training. Command Style = A, Practice Style = B; Reciprocal Style = C; Self-Check Style = D; Inclusion Style = E; Guided Discovery Style = F; Convergent/Divergent Discovery Style = G + H; Learner Designed Individual Program Style = I; Learner Initiated Style = J; Self-Teaching Style = K.

Reproduction teaching styles															
** **	A			B			C			D			E		
	*X* ^2^	df	*p*	*X* ^2^	df	*p*	*X* ^2^	df	*p*	*X* ^2^	df	*p*	*X* ^2^	df	*p*
Years of Service	10.962	8	.204	2.270	4	.685	4.560	8	.803	5.299	8	.725	5.511	8	.702
Academic Training	9.025	4	.049	5.573	2	.049	4.075	4	.396	2.997	4	.558	7.627	4	.047
Production teaching styles															
** **	F			G + H			I			J			K		
	*X* ^2^	df	*p*	*X* ^2^	df	*p*	*X* ^2^	df	*p*	*X* ^2^	df	*p*	*X* ^2^	df	*p*
Years of Service	3.923	4	.417	2.516	3	.472	.486	3	.992	4.146	3	.246	4.097	3	.251
Academic Training	11.148	8	.038	3.682	6	.720	2.602	6	.857	2.489	6	.870	4.204	6	.649

Since Chi-square statistic is significant, contingency coefficient has been carried out to better know the association between the academic training and teachers' perceived use of teaching styles. The perception related to the use of the Command Style presents the highest significant association with academic training (C = .320, *p *= .049) compared to Practice (C = .257, *p *= .049), Inclusion (=.297, *p *= .047), and Guided Discovery Style (=.218, *p *= .049).

Since the value of *X*^2^ is significant for all teaching styles, it can be inferred that the frequency with which styles are used is not equally popular (Table 3). Specifically, the results show a significant prevalence (>50%) of teachers who prefer to use “often” Command (*X*^2^ = 67.542, *p *= .000), Practice (*X*^2^ = 60.530, *p *= .000) and Convergent/Divergent Production Styles (*X*^2^ = 29.916, *p* = .000). As for the Reciprocal and Guided Discovery, although they are often used significantly (*X*^2^ = 34.048, *p *= .000; *X*^2^ = 44–410, *p *= .000, respectively,) there's a large percentage of teachers who use they never, rarely or sometimes. The Self-Check Styles (*X*^2^ = 29.229, *p *= .000), inclusion (*X*^2^ = 27.060, *p *= .000), Learner Designed Individual Program (*X*^2^ = 10.349, *p *= .016) and Learner-Initiated Style (*X*^2^ = 38.494, *p* = .000) are preferably used by teachers sometimes. The least used is Self-Teaching Style (*X*^2^ = 32.904, *p *= .000).

**Table 3:** Responses for data collected by total sample.

In the last month, during PE lessons how many times did you use the style of…								
	Never	Rarely	Sometimes	Often	Almost Always	*X* ^2^	df	Sign.
A	3.6%	25.3%	9.6%	53.0%	8.4%	67.542	4	.000
B	0.0%	0.0%	10.8%	73.5%	15.7%	60.530	2	.000
C	8.4%	10.8%	28.9%	41.0%	10.8%	34.048	4	.000
D	9.6%	30.1%	32.5%	25.3%	2.4%	29.229	4	.000
E	6.0%	22.9%	32.5%	31.3%	7.2%	27.060	4	.000
F	2.4%	34.9%	22.9%	36.1%	3.6%	44.410	4	.000
G + H	8.4%	20.5%	21.7%	49.4%	0.0%	29.916	3	.000
I	12.0%	22.9%	36.1%	28.9%	0.0%	10.349	3	.016
J	4.8%	19.3%	51.8%	24.1%	0.0%	38.494	3	.000
K	30.1%	48.2%	7.2%	14.5%	0.0%	32.904	3	.000

Table 4 presents the responses to the questionnaire of teachers graduated in Motor Science with 0–4 and 5–10 years of service, respectively. Data analysis showed significant differences in the use of all teaching styles in the graduate teacher group with 0–4 years of experience, but not for teachers with 5–10 years of service except for Self-Teaching Style (*X*^2^ = 14.800, *p *= .001). Questionnaire of teacher's with 0–4 years of service responses suggest a significant preference for proposing motor tasks through the style of Command (*X*^2^ = 11.867, *p *= .008), Practice (*X*^2^ = 18.200, *p *= .000) and Convergent/Divergent Production Style (*X*^2^ = 7.867, *p *= .049) for graduate teachers with 0–04 years of service, while the teachers report a significant preference to sometimes use the Reciprocal (*X*^2^ = 14.333, *p *= .006), Self-Check (*X*^2^ = 13.821, *p *= .003) and Inclusion Style (*X*^2^ = 12.667, *p *= .013). Only Guided-Discovery Style (*X*^2^ = 17.667, *p *= .001) and Self-Teaching Style (*X*^2^ = 10.267, *p *= .016) are used significantly less than other styles.

**Table 4:** Responses for data collected by teachers with motor and sports science degree (0–4 and 5–10 years of service).

	In the last month, during PE lessons how many times did you use the style of…								
		Never	Rarely	Sometimes	Often	Almost Always	*X* ^2^	df	Sign.
Teachers with Motor and Sports Science Degree (0–4 years of service)	** **	** **	** **	** **	** **	** **	*** ***	** **	** **
** **	A	0.0%	26.7%	13.3%	50.0%	10.0%	11.867	3	.008
** **	B	0.0%	0.0%	13.3%	70.0%	16.7%	18.200	2	.000
** **	C	6.7%	13.3%	36.7%	36.7%	6.7%	14.333	4	.006
** **	D	10.0%	26.7%	33.3%	30.0%	0.0%	13.821	3	.003
** **	E	6.7%	30.0%	36.7%	23.3%	3.3%	12.667	4	.013
** **	F	6.7%	40.0%	13.3%	36.7%	3.3%	17.667	4	.001
** **	G + H	13.3%	20.0%	20.0%	46.7%	0.0%	7.867	3	.049
** **	I	10.0%	16.7%	36.7%	36.7%	0.0%	6.800	3	.079
** **	J	0.0%	20.0%	53.3%	26.7%	0.0%	5.600	2	.061
** **	K	30.0%	46.7%	10.0%	13.3%	0.0%	10.267	3	.016
Teachers with Motor and Sports Science Degree (5–10 years of service)									
** **	A	0.0%	33.3%	13.3%	46.7%	6.7%	6.067	3	.108
** **	B	0.0%	0.0%	0.0%	73.3%	26.7%	3.267	1	.071
** **	C	0.0%	0.0%	26.7%	60.0%	13.3%	5.200	2	.074
** **	D	0.0%	33.3%	46.7%	13.3%	6.7%	6.067	3	.108
** **	E	0.0%	40.0%	33.3%	20.0%	6.7%	3.933	3	.269
** **	F	0.0%	0.0%	46.7%	53.3%	0.0%	.067	1	.796
** **	G + H	0.0%	0.0%	40.0%	60.0%	0.0%	.600	1	.439
** **	I	13.3%	26.7%	40.0%	20.0%	0.0%	2.333	3	.506
** **	J	0.0%	20.0%	60.0%	20.0%	0.0%	4.800	2	.091
** **	K	6.7%	80.0%	0.0%	13.3%	0.0%	14.800	2	.001

The Higher Institutes of Physical Education teachers' group with 5–10 years of service did not show any statistically significant differences in the use of styles (Table 5). As for the Higher Institutes of Physical Education teachers with >10 years of service, significant differences emerged only in the style of Command (*X*^2^ = 22.739, *p *= .000), Practice (*X*^2^ = 18.087, *p *= .000) and Learner Initiated Style (*X*^2^ = 13.696, *p *= .003).

**Table 5:** Responses for data collected by higher institutes of physical education teachers’ (5–10 and >10 years of service).

	In the last month, during PE lessons how many times did you use the style of…								
		Never	Rarely	Sometimes	Often	Almost always	*X* ^2^	df	Sign.
Higher Institutes of Physical Education teachers’ group (5–10 years of service)	** **	** **	** **	** **	** **	** **	*** ***	** **	** **
** **	A	9.1%	18.2%	9.1%	45.5%	18.2%	4.909	4	.297
** **	B	0.0%	0.0%	0.0%	72.7%	27.3%	2.273	1	.132
** **	C	18.2%	9.1%	27.3%	27.3%	18.2%	1.273	4	.866
** **	D	9.1%	27.3%	27.3%	36.4%	0.0%	1.727	3	.631
** **	E	9.1%	0.0%	18.2%	45.45%	27.3%	3.933	3	.269
** **	F	0.0%	36.4%	18.2%	27.3%	18.2%	1.000	3	.801
** **	G + H	9.1%	27.3%	9.1%	54.5%	0.0%	6.091	3	.107
** **	I	18.2%	27.3%	18.2%	36.4%	0.0%	1.000	3	.801
** **	J	18.2%	18,.2%	36.4%	27.3%	0.0%	1.000	3	.801
** **	K	54.5%	27.3%	0.0%	18.2%	0.0%	2.364	2	.307
Higher Institutes of Physical Education teachers’ group (+10 years of service)									
** **	A	4.3%	26.1%	0.0%	65.2%	4.3%	22.739	3	.000
** **	B	0.0%	0.0%	4.3%	73.9%	21.7%	18.087	2	.000
** **	C	13.0%	13.0%	21.7%	39.1%	13.0%	5.913	4	.206
** **	D	13.0%	39.1%	21.7%	26.1%	0.0%	3.261	3	.353
** **	E	8.7%	17.4%	34.8%	34.8%	4.3%	9.391	4	.052
** **	F	0.0%	47.8%	26.1%	26.1%	0.0%	2.174	2	.337
** **	G + H	8.7%	26.1%	21.7%	43.5%	0.0%	5.969	3	.127
** **	I	13.0%	21.7%	43.5%	21.7%	0.0%	4.652	3	.199
** **	J	4.3%	17.4%	56.5%	21.7%	0.0%	13.696	3	.003
** **	K	30.4%	39.1%	13.0%	17.4%	0.0%	3.957	3	.266

## 4 Discussion

The aim of the present study is to analyze teachers' perception of the use of teaching styles during physical education lessons according to two attributes: years of service and academic training. While years of service are not a discriminating factor in determining significantly whether teaching styles are used, academic training represents an attribute that can influence the frequency with which different teaching styles are used (perception). Specifically, the contingency coefficient reported a significant association between academic training and attendance for Command, Practice, Inclusion and Guided Discovery Styles. So, it is possible to infer that academic training and not years of service is a significant predictor for the variation in teachers' perception use of teaching styles. Moreover, by performing analyses on samples split by years of service and academic training, the results can be summarized as follows.

Command and Practice Styles are those most often used by the total sample and in individual subgroups, regardless of years of service and academic training. However, significant data were highlighted in the Graduate Teachers' group with 0–4 years of service and in Higher Institutes of Physical Education teachers' group with >10 years of service. In fact, the proposal of motor tasks with the style of command and practice allows the teacher to maintain greater decision-making autonomy during activities, which could correlate with (a) the lesser experience of teachers with fewer years of service (0–4), (b) with the will to better manage the class as the age and years of service increase, and (c) the different academic education among graduates and those attending the Higher Institutes of Physical Education. In particular, the third hypothesis seems to be confirmed by the contingency coefficient analysis.

Guided discovery and Convergent/Divergent Discovery Styles are most frequently used by graduate teachers, regardless of seniority. Although these styles are most frequently used by teachers with 5–10 years of service, the results are significant for teachers with 0–4 years of experience. It would therefore seem that among the younger and newly graduated teachers there is a greater tendency to use production styles and thus leave greater decision-making autonomy to the pupils. This hypothesis would be supported by the contingency coefficient for the Guided Discovery Style, but not the Convergent/Divergent Production Style.

The present is—at the best of our knowledge—the first Italian study aiming at assessing PE teachers' perceived use of teaching styles. One of the main important outputs (i) is that academic training can influence and determine a different perceived use of teaching styles during practice. In Italy it is important to consider the innovation process at the end of the nineties that characterized the educational systems of students and universities that led to the transformation of the Higher Institutes of Physical Education in Faculty and Degree Courses in Motor Science (29), as adaptation to the structure and organization of other European Union countries.

It was a cultural passage involving the overcoming of an old legislation that had until that time characterized the Higher Institutes of Physical Education, conditioning the teaching, scientific research and the definition of employment opportunities and professional profile. The new Degree Course highlighted the links and interdependence between theory and practice (designing, conducting, evaluating) that characterize the motor and sports field, the contents and the teaching methods, depending on the competence that the student will have to demonstrate at the end of the curriculum, based on consistent reciprocal relationships between scientific-theoretical evidence and good practices, that is, teaching-educational interventions based on evidence (29–32).

In Italy—as required by the National Guidelines for primary school and the first grade of secondary school—the enhancement of physical literacy is a prerogative and development aim for for PE teachers. Programming for motor skills in the school curriculum involves a significant cultural and methodological turning point that highlights the educational value of motor activities.

The term competence used in the Ministerial Documents entered the teacher's lexicon with the passage from the National Indications, to indicate a planning and an educational action that considered the person in its entirety and a personalized teaching.

Therefore, as part of the teaching of motor activities, the proposal of motor tasks through different organizational modes (individual tasks, in pairs, in small groups, circuits, relay, paths, team games, etc.) is functional to the achievement of the educational objectives set in the specific didactic units. In addition, the definition and acquisition of motor skills, declined in terms of skills and knowledge, allows not only to learn gradually more complex motor skills, but also to develop the skills that allow the child to perform variable movements, skills that can also be transferred and applied in different disciplinary areas and, more generally, in relationship life.

Therefore, the University educational courses should guarantee a more extended and deepened formation on the topics of the didactics of the motor activities, and, in this sense, academic training could represent a key attribute to ensure greater knowledge, mastery, and competence in the use of teaching styles. In fact, a recent study highlighted the preferences about the use of styles according to the teaching degree, showing that Command Style was the most widely used by graduate teachers in Physical Activity and Sport Sciences, while teachers with degree in Physical Education use a wider range of teaching styles (33).

The second meaningful output is that some teaching styles, such as command and practice, are used more frequently than others (ii). These results are in line with those of the study of Constantinides and Orestis Antoniades (34) that have highlighted a significant preference by the PE teachers to use the styles of reproduction, with reference to the styles of command and practice, rather than the production ones. A recent review of literature analyzed the frequency of use of teaching styles according to the Mosston and Ashworth's Spectrum of Teaching Styles in 13 studies from 15 countries with the following results: (a) reproduction styles are used more frequently than production styles, (b) command, practice and inclusion styles are the most widely used, (c), guided discovery and convergent production styles are sometimes used, while (c) the rest of the styles are rarely used (35). The results are confirmed by other studies that confirms the preference for the use of command style, practical and reciprocal, while discovery teaching styles are less used (36, 37). In addition to academic training, another interesting explanation of why teachers prefer reproduction styles can be provided by motivation to teaching. In fact, a study showed that teachers with a high intrinsic motivation more frequently adopt student-oriented approaches and production teaching styles, while teachers not autonomously motivated adopt teacher-centered approach and reproduction teaching styles usually (38). Moreover, recent studies showed that senior PE teachers failed to use a wide range of teaching styles during their lessons (39), preferring the Practice Style due to its ability to facilitate activities and provide feedback, and allowing students to exercise motor skills and receive immediate feedback (40, 41).

## 5 Conclusion

The present study is conducted on secondary school PE teachers who participated in the Regional Observatory of Motor Development and Health Behavior Project. Regarding the sample recruitment, it was applied a convenience sampling, and this could lead to some bias (e.g., sample may not be representative of the reference population). Moreover, due to the small sample involved in this study the invariance test according to gender has not been performed. Despite teachers were given a didactic document describing each teaching style before completing the questionnaire, as confirmed by other studies another important limitation is the lack of knowledge of teaching styles by teachers (26). These two main limitations do not allow us to generalize results.

Future studies should apply more reliable sampling procedure, increase the sample size, extend the study to PE primary, secondary and secondary school teachers, perform invariance test to assess differences according to gender, and provide teacher training and workshop before assessment. Moreover, future research could evaluate the use of teaching styles during the different phases of the PE lesson and compare PE teachers' perception with data collected by external observers.

Scientific research on educational neuroscience and pedagogical reflective practice highlighted the importance to study the teacher's behavior and understand how to promote significant and differentiated motor learning. This will allow to generate interdisciplinary circular processes—theory-practice-theory—to oriented to increase the quality and intentionality of teaching in PE. In addition, the expansion of places of teaching motor skills (school, sport, leisure) and the increased sedentary habits, the uncontrolled use of technologies, have limited the mature reflection on teaching methods that becomes an essential link for the quality of teaching and learning process. In every lesson and in the curricular development, the choice of the modalities through which to organize the didactic setting, opens in the students well defined learning windows that become access ways for the development of disciplinary objectives, interdisciplinary and transversal and for their interactions.

## Data Availability

The raw data supporting the conclusions of this article will be made available by the authors, without undue reservation.
